# Ionic Liquid Applications in Peptide Chemistry: Synthesis, Purification and Analytical Characterization Processes

**DOI:** 10.3390/molecules17044158

**Published:** 2012-04-05

**Authors:** Alesia A. Tietze, Pascal Heimer, Annegret Stark, Diana Imhof

**Affiliations:** 1 Pharmaceutical Chemistry I, Institute of Pharmacy, University of Bonn, Brühler Straße 7, Bonn D-53119, Germany; Email: pheimer@uni-bonn.de (P.H.); dimhof@uni-bonn.de (D.I.); 2 Institute of Technical Chemistry, University of Leipzig, Linnéstraße 3, Leipzig D-04107, Germany; Email: annegret.stark@uni-leipzig.de

**Keywords:** ionic liquids, peptide synthesis, peptide analysis, reaction media

## Abstract

This review aims to provide a comprehensive overview of the recent advances made in the field of ionic liquids in peptide chemistry and peptide analytics.

**Table:** Abbreviations:

[BF_4_]^−^ = tetrafluoroborate	[C_1_C_1_IM]^+^ = 1,3-dimethylimidazolium
[C_1_mim]^+^ = 1-methyl-3-methylimidazolium	[C_2_mim]^+^ = 1-ethyl-3-methylimidazolium
[C_4_C_1_pyrr]^+^ = 1-butyl-1-methylpyrrolidinium	[C_4_mim]^+^ = 1-butyl-3-methylimidazolium
[C_6_mim]^+^ = 1-hexyl-3-methylimidazolium	[C_8_mim]^+^ = 1-octyl-3-methylimidazolium
[C_i,j,k,l_N]^+^ = tetraalkylammonium	[DEP]^−^ = diethyl phosphate
ESI = electrospray ionization	[Et_3_NH]^+^ = triethylammonium
[EtOSO_3_]^−^ = ethylsulfate	[guan]^+^ = guanidinium
[Me_2_PO_4_]^−^ = dimethylphosphate	[MeOSO_3_]^−^ = methylsulfate
[MOEMIm]^+^ = 3-(2-methoxyethyl)-1-methyl-imidazolium	
[N(CN)_2_]^−^ = dicyanamide	[OAc]^−^ = acetate
[OTf]^−^ = triflate	[OTs]^−^ = tosylate
[PF_6_]^−^ = hexafluorophoshate	[SbF_6_]^−^ = hexafluoroantimonate
[SCN]^−^ = thiocyanate	[NTf_2_]^−^ = bis(trifluoromethanesulfonyl)imide
BOP = benzotriazole-1-yl-oxy-tris-(dimethylamino)-phosphonium hexafluorophosphate	
CCA = α-cyano-4-hydroxycinnamic acid	DCC = N,N'-dicyclohexylcarbodiimide
DHB = 2,5-dihydroxybenzoic acid	DIEA = N,N-diisopropylethylamine, or Hünig’s base
DMAP = 4-dimethylaminopyridine	DMED = N,N-dimethyl-ethylenediamine
GSH = glutathione	
GTHAP = [1,1,3,3,-tetramethylguanidinium-2,4,6,-trihydroxyacetophenone]	
H_2_O_2_ = hydrogen peroxide	H_3_PO_4_ = phosphoric acid
HATU = O-(7-azabenzotriazol-1-yl)-N,N,N',N'-tetramethyluronium hexafluorophosphate	
HPLC = high-performance liquid chromatography (high-pressure liquid chromatography)	
IL = ionic liquid	ILM = ionic liquid matrices
kDa = kilodalton	LSER = linear solvation free energy relationship
MPG = 2-methyl-2-(*p*-tolyl)-glycine	MALDI = matrix-assisted laser desorption/ ionization
MS = mass spectrometry	MOPS = 3-[*N*-morpholino]propanesulfonic acid
NCL = native chemical ligation	NMR = nuclear magnetic resonance
OGp = chymotrypsin and trypsin	RP = reversed phase
PyBOP = (benzotriazol-1-yl-oxytripyrrolidinophosphonium hexafluorophosphate)	
SCIL = surface-confined ionic liquids	SCm = V8 protease
SPPS = solid phase peptide synthesis	TFA = trifluoroacetic acid
THF = tetrahydofurane	TLC = thin layer chromatography
TOF = time-of-flight	TRH = thyroliberin (thyrotropin-releasing hormone)

## 1. Introduction

During the last decade the interest in establishing alternative solvents for organic synthesis to provide safe and environmentally clean processes has increased dramatically. Alternative solvent media that can provide suitable or even better reaction conditions for organic synthesis and which are less hazardous than conventional organic solvents are “molten salts” or so-called ionic liquids (ILs). Numerous reports have been published describing the unique properties of ILs such as negligible vapor pressure, low melting point, special phase behavior, electric conductivity, low flammability, and high thermal stability. The applications range from transition metal-mediated catalysis, e.g., hydrogenation, oxidation, or hydroformylation, to organic reactions such as the Diels-Alder reaction, the Friedel-Crafts reaction, cycloaddition, and esterification [1,2,3]. In addition, ILs became very attractive in a number of fields involving biological macromolecules, e.g., enzymes and proteins, where they lead to structure stabilization [4] and are applied to biotransformations by enzymatic catalysis [5], protein crystallization [6], and fluorescence quenching immunoassays [7]. In other applications, ILs show advantages due to their favorable solvation properties for polar substrates, including carbohydrates [8,9], proteins [10], and reduced emissions, but also as solvents in analytical chemistry and in chemosensing strategies [11].

In comparison to conventional solvents an IL consists of an anion and a cation, *i.e.*, it possesses a dual character. For this reason, ILs are salts that can provide a suitable medium for biomolecules. Depending on the ionic liquid composition, the solvation environment, they can be adjusted to fit a specific solute and process requirements, such as solubility or phase separation from an organic or an aqueous phase [12]. By manipulating the ionic solvent properties it is possible to design ILs for special reaction conditions, which in case of enzymatic reactions affect, for example, reaction rate, enzyme selectivity, and substrate solubility [5]. Lately, several reports about the interactions between an ionic liquid and various biomolecules were published. However, the nature and the forces of IL-solute interactions are still too little understood, to answer the questions why ILs stabilize the active form of enzymes/proteins [8,13], or why they provide an ideal platform for the folding of cysteine-rich peptides [14]. In the coming years, these will become more and more attractive in the field of biochemical research.

In 2008 [15], Plaquevent *et al*. presented an excellent overview of IL applications in amino acid and, in part, peptide chemistry, as well as of the use of IL components to produce novel amino acid-based ILs [16,17]. Their intention was to answer the following quite comprehensive questions: Is it possible that ILs can provide a better reaction medium than traditional organic solvents and in which fields of chemistry and biochemistry can they be applied? However, there are still no satisfying answers and many more questions are open due to the large variety of applications offered in this field of research. During the last three years, interest in the use of ILs in peptide and protein research increased immensely. Finding new ways to improve coupling reactions, solubility of peptides and proteins in aqueous/buffer solutions, and their chemical characterization occupied the mind of peptide, protein and analytical chemists. Hence, detailed studies were carried out, including investigations on the influence of peptide/protein concentration on the outcome of a biochemical reaction, the effect of the ionic liquid on the peptide/protein structure, the effect on the function of a peptide/protein of interest, and more generally, on how to increase the stability and reactivity of such biomolecules in ILs. 

The main challenge is to select the best IL for a particular process and/or application. This review intends to support advances made in the field of IL applications in peptide chemistry by summarizing the current state in the field and condense the information available in the literature. Indeed, one may ask the question, why we are focusing our attention on peptides? Polypeptides and proteins are present in all living cells in a rather large number and variety. Model peptides are usually used to understand functions of large proteins [18]. However, it is also generally accepted that the effects and behavior of peptides in various reaction media differ from those of proteins. Proteins usually fold to form particular three-dimensional shapes, which determine their actions, while polypeptides are structurally less constrained, *i.e.*, they can adopt many conformations in solution. Thus, proteins possess a rather well-defined tertiary structure that is in many cases missing in the case of peptides. Therefore, we intend to give an overview of the new fields of investigations that appeared during the last years with a focus on the use of ionic liquids in peptide chemistry, including purification, and analysis. Also, this survey represents new trends, which have appeared in the literature following the review of Plaquevent *et al*. [15] four years ago, to help to choose a suitable IL for the desired reactions and characterization techniques.

## 2. Ionic Liquids in Peptide Chemistry

In this part, we describe the applications of ILs in peptide chemistry: peptide synthesis and modifications of special peptides. We included different synthetic strategies of conventional methods, where organic solvents are used primarily, but focused our attention on ILs as reaction media and the advantages and disadvantages of their use in peptide synthesis. Our report highlights the achievements made in peptide synthesis since the publication of the review mentioned earlier [15].

The choice of the ionic liquid in a peptide-chemical reaction depends on various factors, but most importantly is dependent on: (a) the solubility of the peptide/protein and/or its precursors and (b) the melting point of the ionic liquid. The latter aspect is important if the reaction is to be conducted in the absence of any co-solvent such as water, which would reduce the viscosity of the reaction mixture, but also affect the peptide-ionic liquid interactions. In analogy to other biopolymers, such as cellulose or silk, ionic liquids with rather nucleophilic anions dissolve proteins due to their ability to break the inter- and intramolecular hydrogen bonding network. Similarly, ionic liquids reduce the ability to form peptide-peptide hydrogen bonds, thus lowering the risk of protein denaturation through misfolding and aggregation. However, due to this property, protein-dissolving ionic liquids often feature melting points well above room temperature, which reduces their potential for applications with thermolabile peptides and proteins. While the anion is involved directly in the dissolution process, the cation choice can be used to modulate the properties of the ionic liquid with regards to viscosity, melting point and therefore mass transfer limitations somewhat. Dialkylimidazolium-based ionic liquids belong to the best investigated group of ionic liquids. However, it needs to be noted that questions of toxicity of ILs arise, especially if the peptide is to be used as pharmaceutical. For example, it can be expected that thiocyanide-, methylsulfate or biscyanamide-based ionic liquids will be toxic, while chlorides, sulfates and acetates should be less or non-toxic. In fact, [C_2_mim][OAc] is non-toxic and has been proven to be the IL of choice for the dissolution and conversion of the linear, rather hydrophobic peptides [14].

### 2.1. Peptide Assembly

**Figure 1:**
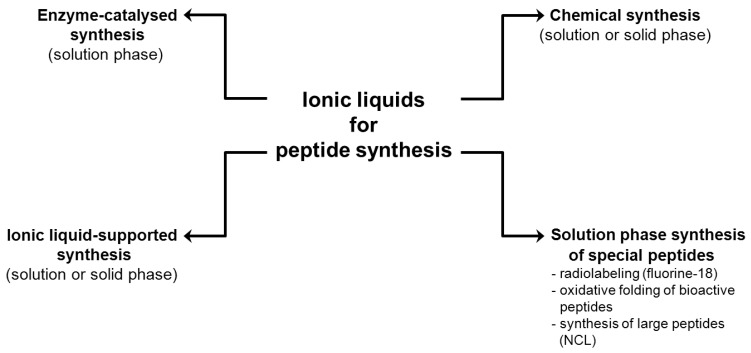
Schematic representation of ionic liquid-applications in peptide synthesis.

Nowadays, a large variety of methods is available to produce or obtain peptides, including isolation from natural sources (e.g., extraction from plants, animals and other organisms), recombinant synthesis, and production by chemical or enzymatic synthesis [19,20,21,22,23]. Only a few manuscripts have reported that ILs can be applied in this field of peptide assembly as pure solvents, as co-solvents in aqueous systems or as biphasic systems [8,24], in both, solid and solution phase synthesis as well as in enzyme-catalyzed peptide synthesis (Figure 1). 

In general, the choice of the synthetic method is usually determined by the length and the quantity of the peptide of interest. For example, enzymatic peptide synthesis is applied for the production of small peptides, e.g., di- or tripeptides [25], to obtain large quantities in industrial production scale. This method benefits from the high specificity of the reaction, the avoidance of side chain protection of the substrates and the racemate-free formation of the products. Chemical synthetic protocols have focused firstly, on the synthesis of longer peptides by Merrifield solid phase peptide synthesis (SPPS) [26], which is limited to a length of approximately 50 amino acids (though longer peptides can be prepared depending on the primary amino acid sequence), primarily because of the steric hindrance and accumulation of undesired and truncated sequences during chain prolongation. Secondly, for the synthesis of polypeptides (>50 amino acids) or small proteins the method of segment ligation has been introduced as an alternative for recombinant production (see Section 2.2.3). The combination of chemical and enzymatic synthesis has been described by Guzman *et al*. representing a method that might be quite suitable for selected peptides [23]. In other procedures, the use of a soluble polymer support, which is called “liquid-phase” peptide synthesis, has been demonstrated [27]. This method was successfully applied for the synthesis of several oligopeptides, but the limiting factor here usually is the solubility during peptide assembly and work-up, in particular in solvents such as water or diethylether. In addition, “fluorous-phase” synthesis was established, where fluorous supports (*i.e.*, trialkoxybenzhydryl-type, Wang-type and *tert*-butyl-type support) and fluorous chemistry were used for peptide synthesis exemplified for bioactive peptides, e.g., TRH [28,29]. However, the most common procedure of peptide synthesis still remains SPPS.

However, the methodologies applied nowadays clearly suffer from limitations covering solubility, low selectivity and yield, reactivity and steric issues as well as rather high costs of reagents. Thus, there is a high demand for new and/or optimized methods in this field. Indeed, ionic liquids have found their applications in the field of peptide chemistry in the last decade (Table 1). Since it was noticed that ILs improve the solubility of different reactants, separation of the products and the recovery of the ILs was possible [8,9,10,11,12,13,14,15]. ILs became more and more popular in peptide chemistry as summarized in the following sections. 

**Table 1:** Summary of the ionic liquids used in peptide chemistry.

Ionic liquid	Abbreviation	Application	Ref.
1-methoxyethyl-3-methyl-imidazolium hexafluoro-phosphate or tetrafluoroborate	[MOEMIM][PF_6_] [MOEMIM][BF_4_]	Enzymatic peptide synthesis	[30]
1-butyl-3-methylimidazolium hexafluorophosphate	[C_4_mim][PF_6_]	Chemical peptide synthesis	[15,31,32,33,34]
1-hydroxyethyl-1-methyl-imidazolium tetrafluoroborate 3-(2-hydroxyethyl)-1-methyl-imidazolium tetrafluoroborate	-	IL supported peptide synthesis	[35,36]
1-butyl-3-methylimidazolium X	[C_4_mim][X] (X = BF_4_, PF_6_, SbF_6_, OTf, NTf_2_)	Radiolabeling of peptides	[37,38]
1-ethyl-3-methylimidazolium X 1-butyl-3-methylimidazolium acetate	[C_2_mim][X], (X = OAc, DEP, OTs, N(CN_2_)_2_) [C_4_mim][OAc]	Oxidative folding and native chemical ligation of cysteine-containing peptides	[14,39]

#### 2.1.1. Enzymatic Peptide Synthesis

Some reports describe ILs as suitable media to increase the solubility, stability and activity of enzymes. [10,13,40,41,42,43] For example, Constantinescu *et al*. [41] studied the denaturation of enzymes in ILs including the cations [C_2_mim]^+^, [C_4_mim]^+^, [C_6_mim]^+^, [C_4_C_1_pyrr]^+^, [C_i,j,k,l_N]^+^, [guan]^+^ and the anions [SCN]^−^, [MeOSO_3_]^−^, [EtOSO_3_]^−^, [OTf]^−^, [NTf_2_]^−^, [N(CN)_2_]^−^. It was found that the stability at higher temperature (70 °C) was increased compared to aqueous buffer solution, with [C_i,j,k,lN_]^+^ and [NTf_2_]^−^ showing the best results. These studies were later extended by others, for example to the use of [C_2_mim][FSI] (FSI = bis(fluorosulfonyl)imide) for the α-chymotrypsin-catalyzed reaction of Ac-Trp-OEt with Gly-Gly-NH_2_ to yield Ac-Trp-Gly-Gly-NH_2_ (see also below) [42]. Here, a 16-fold increase in the initial rates for peptide synthesis was observed compared to the reaction carried out in acetonitrile. In addition to these findings, they also found a 17-fold higher activity for α -chymotrypsin in ILs compared to organic solvents. 

Besides, other applications of ILs have been reported with respect to proteins, e.g., Summers and Flowers have described the renaturation of proteins in the IL ethylammonium nitrate as a one-step process [44]. This IL prevented aggregation and caused the refolding of proteins, e.g., lysozyme, resulting in its active state with up to 90% of active protein [44]. ILs as suppressors of protein aggregation and useful supports for refolding processes of denatured proteins were also described by Buchfink *et al*. in 2010 [45]. A series of [C_2_mim]^+^ salts was used with a focus on the impact of the anions as refolding enhancers as well as different model proteins, among them recombinant plasminogen activator rPA. It was found that IL co-solvent systems with an intermediate capacity to solubilize proteins are effective as refolding additives, *i.e.*, were found to permit effective renaturation of rPA. The most promising refolding enhancer in this respect was [C_2_mim][Cl]. By means of fluorescence correlation spectroscopy Sasmal *et al*. found that the presence of 6 M guanidine hydrochloride helps a protein, e.g., human serum albumin, to refold after addition of 1.5 M [pmim][Br] [46]. 

An interesting contribution quantifying the biomolecular interactions of the functional groups in proteins with biocompatible ILs, e.g., diethylammonium and triethylammonium acetate, has been reported recently [47]. Herein, transfer free energies (ΔG’_tr_) of compounds such as Gly, Gly-Gly and cyclo(Gly-Gly) from water to aqueous ionic liquid solutions were determined depending on the IL concentration. The solubility in aqueous solutions decreased with increasing IL concentration (salting-out effect). This was attributed to the non-favorable interactions between IL and individual amino acid residue(s). However, overall all ILs could stabilize the structure of the model compounds—with triethylammonium acetate being the strongest stabilizer among the ILs used. On the other hand, a negative contribution (ΔG’tr) of the peptide backbone from water to higher concentration of e.g., TMAA (50–70%) indicated that the interactions between the solvent and backbone unit are favorable. 

The first attempts in peptide bond formation by enzymes, however, were already reported by Erbeldinger *et al*. in 2000 [31]. Here, the synthesis of *Z*-aspartame in a thermolysin-catalyzed reaction in [C_4_mim][PF_6_] was demonstrated. Upon addition of 5% of water a biphasic reaction mixture was formed, and water exhibited a stabilizing effect on thermolysin. Also, the activity of the enzyme was dependent on the water content. In addition, an increased solubility was observed for the substrate and product upon addition of water. The reaction was monitored by HPLC after mixing of the reaction mixtures with 60% acetonitrile/water containing 10 mM orthophoshoric acid prior to injection. For the final procedure, however, a step allowing for the recycling of the IL was suggested after the reaction occured. The yield of the product was 95%, *i.e.*, the same as found for organic solvents, e.g., ethyl acetate/water mixtures.

In 2007, Xing *et al*. reported the use of an α -chymotrypsin-catalyzed synthesis of protected tripeptides, e.g., the fragment Z-Tyr-Gly-Gly-OEt of Leu-enkephalin, in six different 1-alkyl-3-methylimidazolium hexafluorophosphates and tetrafluoroborates [30]. Again, it was found that the water content has a great influence on the two main factors, enzyme activity and coupling yield. At higher water content, the product is hydrolyzed, thus reducing the reaction yield. However, a small amount of water was essential for the reaction to succeed, *i.e.*, for hydrophobic [MOEMIM][PF_6_] the minimum content was 3%, while for the more hydrophilic [MOEMIM][BF_4_] 8% turned out to be favorable. The authors also reported that [MOEMIM][PF_6_] was the most suitable IL among the ILs tested, and peptides were prepared in 68–75% yield. Further enzyme-supported reactions in ILs have been reported by Wehofsky *et al*. [48] and are in detail in Section 2.2.3, because larger peptide molecules prepared by ligation reactions are in the focus of that study. However, enzymatic acylation of smaller peptides has also been described by other groups, as can be exemplified by the lipase-catalyzed acylation of Lys-Ser x HCl with oleic acid carried out in either organic media (2-methyl-2-butanol) or an ionic liquid ([C_4_mim][PF_6_]) [49]. Substrate conversion in this case was found to depend on peptide solubility which was improved in the IL. Primarily chemoselective acylation occurred at the N_ε_-amino group of lysine, while the side chain acylated serine derivative was not detected at all. Another example came from Malhotra *et al*., who reported on the immobilized protease-catalyzed synthesis of peptides, e.g., Boc-Leu-Trp-OEt, in the ionic liquids [C_4_mim][PF_6_], [C_4_mim][BF_4_], and [C_2_pyr][BF_4_] [50]. Here, product yields were comparable to those obtained in organic solvents, *i.e.*, a maximum yield of 49% for Boc-Leu-Trp-OEt was obtained.

In summary, 1,3-disubstituted imidazolium-based ionic liquids were successfully applied as additives to enzymatic syntheses. They were described to have a positive effect on enzyme-catalyzed coupling reactions, to increase the enzyme’s activity, the coupling yields, the solubility of reagents as well as the product. Interestingly, the necessity of water for optimal coupling yields has been demonstrated regularly. Obviously a minimum amount of water (3–8%) is favorable for the activation respective enzyme.

#### 2.1.2. Chemical Synthesis of Peptides

##### 2.1.2.1. Condensation of Free Amino Acids in Solution

In a symposium report, Smith *et al*. demonstrated for the first time the peptide bond formation, e.g., for the generation of cyclopeptides, in ionic liquids at high temperatures (>100 °C). The thermally stable 1-butyl-3-methylimidazolium hexafluorophospate was used, while water was absent in the reaction mixture. Surprisingly, at temperatures below 100 °C no reaction occurred [32]. Subsequent works also reported the condensation of amino acids in ILs, in particular in 1-butyl-3-methylimidazolium hexafluorophosphate [15,33,34,51]. Here, high coupling yields were achieved for quaternary α-amino acids that are much more difficult to couple than tertiary amino acids [15]. The reaction is thought to proceed through the stabilization of a charged species in the ionic solvent. Interestingly, tetra-, octa- and cyclooctapeptides were obtained in high yields (80–90%), which was comparable to organic solvents (e.g., THF). However, the crude peptides had much higher purities compared to classical organic synthetic protocols. A stabilization effect of the coupling reagents HATU and BOP in the ionic liquid was suggested to be the cause of this observation [34]. Still, finding a suitable extraction protocol for separating the product from the reaction mixture provides rather difficult, but in another publication good isolated yields (66–87%) were obtained by extracting of a protected dipeptide (Z-Gly-MPGOMe) [33,51]. In addition to these reports, Petiot *et al*. described the use of a new ammonium-based poly(ethyleneglycol)-based ionic liquid (PEG-IL) and its use for the synthesis of various dipeptides containing Phe, Gly, Ala, Leu and Glu under microwave activation [52]. As an example, the formation of Boc-Phe-Phe-OMe by using HATU as coupling reagent (3.3 equiv.) in a bis-PEG_350_-IL carried out at 65 °C for 2 h yielded 64% of the product. It was discussed that the polar nature of bis-PEG350-ILs may have a beneficial effect on the solubilization of the starting material, *i.e.*, amino acids and coupling agents. In addition, higher purities of the dipeptides obtained after work up, and reduced reaction times compared to previously reported peptide syntheses in ionic liquids were observed in this study.

As demonstrated, first attempts have been undertaken to synthesize small peptides in ionic liquids as reaction media. In addition, the idea of a direct coupling of an amino acid to form a peptide bond by simply dissolving it in an ionic liquid and thus avoiding protection/deprotection steps seems promising for future applications of ILs in peptide synthesis [15].

##### 2.1.2.2. Ionic Liquid-Supported Amino Acid Condensation

**Figure 2:**
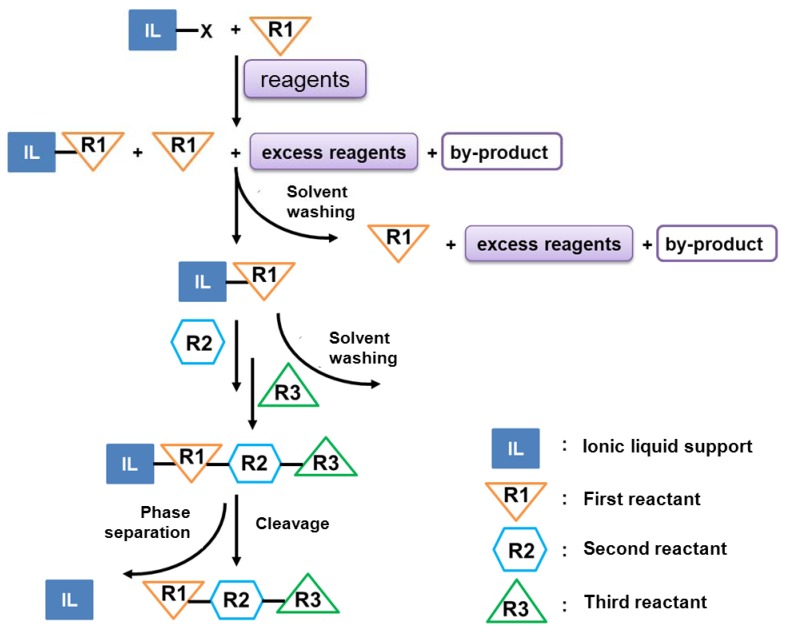
Scheme of ionic liquid-supported peptide synthesis (modified from [36]).

Solid phase peptide synthesis (SPPS) may have several limitations, e.g., solvation of reagents, slow coupling rates, difficult access of the reagents to the reaction sites, and nonlinear reaction kinetics. As a consequence, “liquid phase” methods were developed and applied recently (Section 2.1). One of these methods represents the IL-supported peptide synthesis introduced by Miao and Chan in 2005 [36]. Here, substrates were directly attached to the respective IL, *i.e.*, 3-hydroxyethyl-1-methylimidazolium tetrafluoroborate, and by-products and excess reagents were removed from the desired product and intermediates by using simple washing steps (Figure 2). This synthetic procedure was used to produce Leu^5^-enkephalin of the sequence Tyr-Gly-Gly-Phe-Leu-OH. There were several advantages of this method, including (i) only a low excess of the coupling reagent (two equivalents PyBOP or DCC) was required for the coupling steps instead of commonly used four to five equivalents, (ii) intermediates were easily purified by washing steps (liquid/liquid phase separation), and (iii) purity determination and structure identification were possible through spectroscopic methods (MS, NMR). 

**Figure 3:**
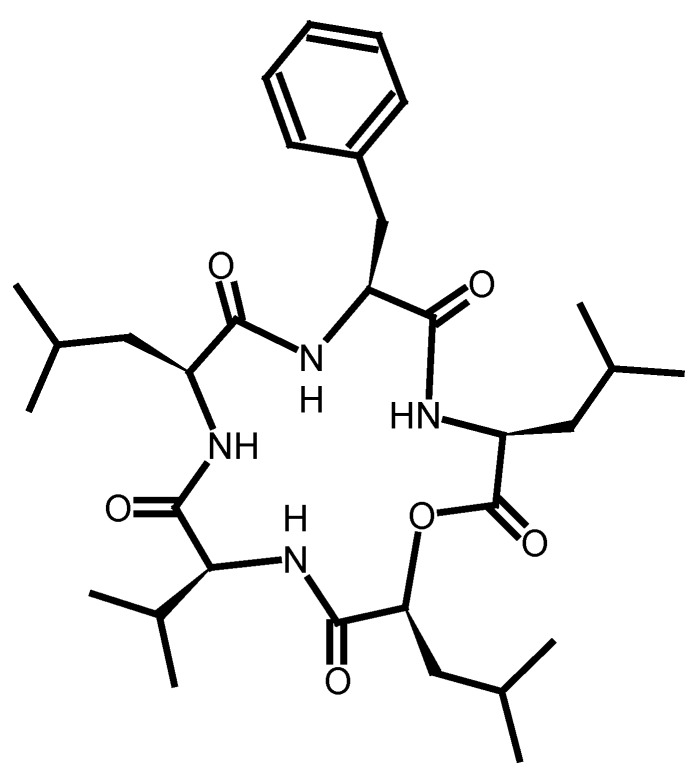
Molecular structure of cyclic depsipeptidesansalvamide A (modified from [57]).

Furthermore, quantitative condensation of the first amino acid with DCC/DMAP and the second residue by using PyBOP/DIEA was achieved [36]. Additionally, IL-imidazolium attached oligomers were used as a soluble/solid support for the synthesis of hexapeptide building blocks. As an example/model the natural peptide Mucin4 containing a high number of serine and threonine residues was chosen. In contrast to previous studies, a mixture of HOBt/HBTU was used as coupling reagents, because of the difficulties to remove tripiperidinophosphine oxide from PyBOP during purification steps. Purification of the peptides was carried out by simple washing steps using water, because peptides were insoluble in water, while the soluble ionic supports could be washed out easily [53]. Shortly after this report, the same research group outlined the use of IL-attached oligomers for the large scale production of oligopeptides, oligosaccharides and oligonucleotides in a patent [35]. Other groups functionalized the ILs, namely cations 1-methylimidazolium and pyridinium and anions bromide, chloride, iodide and tetrafluoroborate, with amino, alcoholic or carboxylic groups. Rather high loadings (2.5–5.0 mmol/g) of the substrates of the ILs were obtained in these studies [54,55]. Additionally, in the review mentioned earlier [15,56], the use of ILs based on histidinium salts for the synthesis of dipeptides was emphasized. They described coupling efficiencies of 78–83%, while no racemization occurred and the required product, the histidinium-coupled alanine derivative, was successfully obtained. The first total synthesis of a bioactive peptide, sansalvamide A (Figure 3), by ionic liquid-supported peptide synthesis was demonstrated by Chen *et al*. [57] Here, final ring closure was achieved by using PyBOP as coupling reagent.

An interesting approach, which combines advantages of SPPS and liquid phase reactions, represents the use of [58] onium salt soluble supports containing a trimethylammonium cation for peptide synthesis of small peptides (e.g., tripeptides). The onium salts-based soluble supports were easily prepared by classical synthetic routes in high yields (74–99%) with a final loading capacity between 1.6 and 3.8 mmol/g, *i.e.*, only 26–50 g per liter solvent was required in order to achieve the traditionally used 0.1 M concentrations during synthesis. Tripeptides were obtained in good yields (98% over two steps). In addition, several positive effects were observed, such as less than 5% diketopiperazine formation, less than 1% racemization for model peptide Val-Leu-Ala, compatibility with conventional reagents, e.g., coupling reagent HBTU or cleaving agent HPF_6_ in methanol, purification of peptides by extraction with water, and recycling of the support. The purification step, for example, could easily be achieved, since the peptide was soluble in water, while onium salts derivatives were soluble in dichloromethane. In addition, reactions could be monitored by NMR and/or HPLC without cleavage from the support rendering this method suitable for high throughput and large scale preparation of peptide libraries [58].

Finally, Cho *et al*. reported the preparation of ionic liquid resins with an ionic liquid environment on solid support by immobilizing ionic liquid spacers on a polystyrene (PS) resin [59]. The properties of IL resins were dramatically changed as the anions of IL were exchanged. The performance of IL resins for solid-phase peptide synthesis (SPPS) was evaluated by measuring coupling kinetics of the first amino acid, and synthesizing several peptides on IL resins.

In our opinion, the coupling of amino acids in ILs can obviously be carried out at a high rate without the use of a large excess of coupling reagents, and this, in turn, is one big advantage of the use of ILs in peptide chemistry.

### 2.2. Peptide Modifications

In this part we describe the progress made with respect to reactions other than the general peptide assembly methods, e.g., the synthesis of radiolabeled peptides, the formation of disulfide bridged polypeptides by oxidative folding and different ligation procedures used to obtain oligopeptides and small proteins. This novel and, at the current state, sparsely investigated research field hints to the successful use of ILs for such processes and opens exciting new ways for the optimization of protocols that are hampered by many drawbacks of conventional methods applied thus far.

#### 2.2.1. Radiolabeling of Peptides

Radiolabeling is an established technique with many applications in biological research, such as the investigation of the distribution of labeled markers *in vivo* (e.g., in humans and in animals) by positron emission tomography [60]. The synthesis of the radiolabeled bioactive compounds, in particular peptides, is usually time consuming and requires complicated procedures and special equipment to purify the product from unwanted non-radioactive and radioactive byproducts. Strictly anhydrous conditions are highly recommended, for example, for fluoride ion displacement reactions in the conventional protocols.

**Figure 4:**
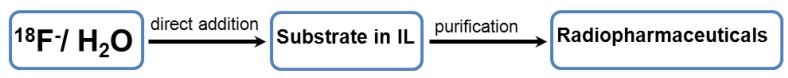
Radio-fluorine-18-labeling performed in an ionic liquid (modified from [37]).

A new approach in this respect has been proposed by Kim *et al*. in 2003 (Figure 4) [37]. Herein, fluorine-18 labeling of alkyl mesylate or alkyl halides, e.g., 2-(3-methanesulfonyloxypropoxy)-naphthalene, in ILs containing a hydrophobic cation ([C_4_mim][X], where X = [BF_4_]^−^, [PF_6_]^−^, [SbF_6_]^−^, [OTf]^−^, [NTf_2_]^−^) was described for the first time. This method was successful despite the addition of a small amount of water, which is in contrast to the conventional method used. The ILs enhanced the reactivity of fluorides and also, a reduction of by-product formation (alkenes, alcohols) was observed. This approach led to further developments in this field as reported by Schirrmacher *et al*. [38]. The IL [C_4_mim][OTf] was useful for the labeling of short model peptides, e.g., GSH [38]. Thus, radiolabeling performed in IL (1,3-dialkylimidazolium salts) obviously occurs without the need for complicated procedures and strictly anhydrous conditions, but by direct addition of the IL to the reaction mixture and further simple purification steps.

#### 2.2.2. Disulfide Bond Formation and Peptide Folding

Establishing efficient strategies for the synthesis of cysteine-rich peptides has always been a challenging task in the past. Generally, the incorporation of cysteine residues in peptide sequences is difficult as it requires the protection of the highly reactive thiol group [18,61,62,63]. Cysteine, however, is very important since many naturally occurring peptides contain intramolecular disulfide bonds that stabilize the biologically active conformation [18,64,65]. Thus, one application of cysteine-residues in peptide chemistry represents disulfide bridge formation that may be achieved at various stages of the synthesis, on-resin as well as in solution [62,66,67,68]. Protecting group strategies differ according to the number of cysteine residues and disulfide bonds to be formed. While peptides containing one disulfide bridge are mainly prepared by oxidation (e.g., air, H_2_O_2_, iodine) in solution at low concentrations (10^−3^ to 10^−4^ M), for peptides with two or more disulfide bridges the situation is more complicated since intramolecular mispairing (scrambling) and/or intermolecular dimerization or oligomerization may occur [61,62,63].

**Figure 5:**
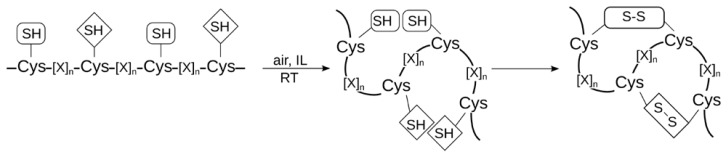
Oxidative folding of cysteine-rich peptides performed in ILs.

The major disadvantages of these conventional methods are: (i) the requirement for a high dilution of the reaction mixture, (ii) very long reaction times, (iii) inadequate solubility of hydrophobic peptides in buffer solutions (including the need for organic solvents: isopropanol, methanol, acetonitrile), and (iv) the difficulty to control oxidation that requires the reaction to be carried out at low temperatures (usually 4 °C). Consequently, there are huge problems for producing such peptides in large amounts and sufficient purity. Over the years, many scientists were searching for a method that can overcome these problems. In our own work [14], we recently described for the first time the use of 1-ethyl-3-methylimidazolium-based ILs with different anions including small hydrogen-bond acceptors (acetate [OAc]^−^, diethyl phosphate [DEP]^−^) as well as larger, less-coordinating anions (tosylate [OTs]^−^, dicyanamide [N(CN)_2_]^−^) for the oxidative folding of neuropeptides (Figure 5). 

A very good solubility of hydrophobic peptides, e.g., δ-EVIA and δ-SVIE, containing six cysteine residues was observed in these ILs. The reaction was performed in ILs of low water content (between 0.5–3%), in a very small volume and a rather high concentration of the peptides (10–15 mM) [14]. Thus, this seems to be a promising method for up-scaling the production of cysteine-rich peptides, also because the formation of by-products and misfolded species was minimized in comparison to conventional methods (oxidation in buffer solution [69,70]). In contrast to the latter, there is no need for additional redox reagents, thus the reaction can be performed in the pure IL. A stabilization of pre-formed secondary structures of the peptides investigated by the ILs was suggested to be the cause of these observations.

Later, different naturally occurring cysteine-rich peptides were studied to understand the nature of interaction between ILs and peptides. Various model peptides—short (10 amino acids) and medium-sized (20–40 amino acids) native peptides with varying numbers of cysteine residues in their structure (from two to six) were subjected to these investigations. Depending on the net charge, amino acid content and hydrophobicity of the peptide, the efficiency in the ILs used differed strongly. In contrast to conotoxins µ-SIIIA, µ-PIIIA, δ-EVIA, and δ-SVIE that showed best results when using [C_2_mim][OAc] as reaction medium [14], optimum conversion of linear CCAP-vil [39] was observed in [C_2_mim][OTs] (yield >90% according to HPLC analysis) and to a lesser extent in [C_2_mim][DEP] (yield >80% according to HPLC analysis). Interestingly, the target peptide did not form the oxidized version in [C_2_mim][OAc] in acceptable yields (57%), and the yield was even lower in case of [C_2_mim][N(CN)_2_] (approximately 44%). When increasing the lipophilicity of the ionic liquids (by increasing the alkyl substituent from [C_2_mim][OAc] to [C_4_mim][OAc]), the yield of the oxidized product decreased to approximately 47% [39].

**Figure 6:**
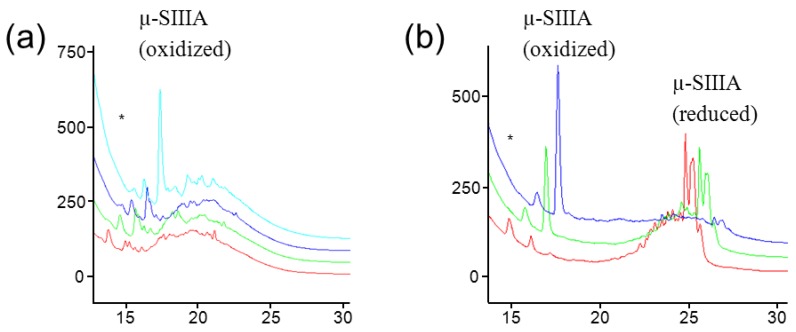
RP-HPLC elution profiles of µ-SIIIA (crude mixtures) after oxidative folding in [C_2_mim][OAc] depending on (**a**) water content (cyan with 3% water, blue—5%, green—7.5%, red—10%) and (**b**) temperature (blue curve—80 °C, green—60 °C, red—40 °C). * Peak from ionic liquid.

It is clear from these studies that oxidative folding depends on the proper choice of the ionic liquid. Also, peptides with a high net charge are apparently more efficiently oxidized in ILs containing more basic anions, such as [C_2_mim][OAc], compared to small uncharged peptides such as CCAP-vil, where the most suitable ionic liquid contains a less basic anion, *i.e.*, [C_2_mim][OTs]. Unpublished data related to our former work showed interesting dependencies of the reaction outcome and product purities on water content and reaction temperature (Figure 6). This can be exemplified for the conopeptide µ-SIIIA, where already an increase of the water content from 3-10% was disadvantageous for product formation. In contrast, an increase in temperature up to 80 °C was favorable for the oxidation reaction.

The role of ILs in oxidative folding of peptides is still sparsely investigated, since further candidates are necessary to derive a scientific explanation for these observations, yet the application of ILs for this reaction clearly has the potential to be developed as a key technology in peptide chemistry in the future. However, an interesting aspect with respect to the folding behavior of peptides was recently presented by Huang *et al*., who reported on the investigation of the folding of model peptides in neat ionic liquids by CD spectroscopy [71]. This study was motivated by the fact that previous IL studies have almost exclusively focused on large functional proteins, such as lysozyme, ribonuclease A and human serum albumin. Indeed, the authors were correct in stating that the interpretation of the results obtained for proteins are complicated by their size and the different degrees of freedom available to either a peptide or a protein. Thus, they focused on small peptides, e.g., α -helical AKA_2_, miniprotein Trp-cage (contains e.g., α -helical part and 3_10_ helix), and Trpzip4 (β-hairpin), in order to understand the effect of ILs, in this case [C_4_C_1_pyrr][NTf_2_], on well-designed secondary structures. The model sequences used represent optimized native folds in an aqueous environment. Huang *et al*. characterized the thermal transitions of these peptides using circular dichroism (CD) spectroscopy and found that, in contrast to the aqueous data, the far-UV CD spectra of AKA_2_ and Trp-cage in [C_4_C_1_pyrr][NTf_2_] indicated only minimal helical structure at low temperatures. However, in both cases, this data and the temperature-dependent far-UV CD spectra suggested apparent heat-induced structure formation with structure persisting to the highest temperatures recorded (92 °C). In contrast to the results of the helical peptides, the CD data for Trpzip4 indicated huge destabilization by the IL medium with increasing temperature [71]. In addition to this report, Debeljuh *et al*. studied the impact of triethylammoniummesylate (TeaMS) on the structure of the Abeta(1–40) peptide for Alzheimer’s disease [72]. They found that the IL was able to induce a conformational change and also that this structural change influences the self-assembly of the peptide into amyloid fibrils. In our opinion, these studies are an important starting point for further investigations on peptide conformations and peptide folding observed in ionic liquids.

#### 2.2.3. Ligation Reactions in Peptide Synthesis

**Figure 7:**
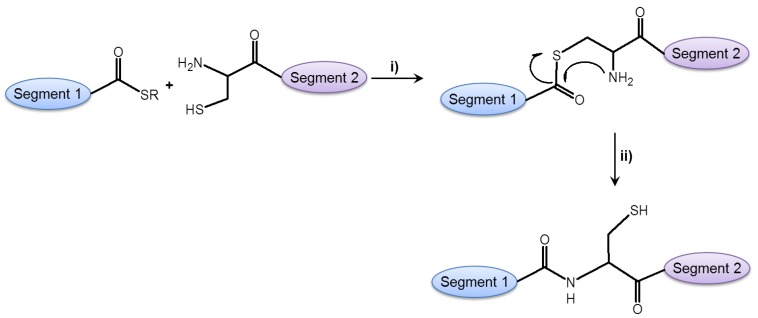
Native chemical ligation occurs between two unprotected peptide segments: (i) transthioesterification; (ii) S to N acyl transfer [73,74]).

Chemical ligation reactions are useful tools for the assembly of small to medium-sized proteins and protein domains starting from synthetic peptide fragments [73,74,75]. This type of chemical protein synthesis allows the modification of the molecular structure offering new insights into protein functions. Though 50 to 100-mer peptides can be assembled on a solid support, synthesis of longer peptides and proteins by the Merrifield method is often limited (see section 2.1 and section 2.1.2). Methods of segments ligation have been developed primarily in order to circumvent limitations in chain length. These segment condensation reactions, on the other hand, may be hampered by the low solubility of peptide fragments for the condensation reaction carried out in water or aqueous/organic solvent mixtures, and the stability of biomolecules. Both aspects are very important for the ligation reaction itself, and have an impact on later purification steps. One of the most widely used methods in this respect is the so-called “Native Chemical Ligation” (NCL) introduced by Kent and coworkers [73,74,75]. Here, an unprotected peptide with a thioester at the C-terminal and an unprotected peptide with an N-terminal cysteine react to yield a peptide bond. The mechanism of this reaction is shown in Figure 7. Related methods are the Staudinger ligation, which occurs between a peptide azide and a phosphinothioester, and protease-catalyzed ligations, where specific peptide esters (e.g., substrate mimetics) react regioselectively and stereospecifically with nucleophilic peptide species [73,76,77].

**Figure 8:**
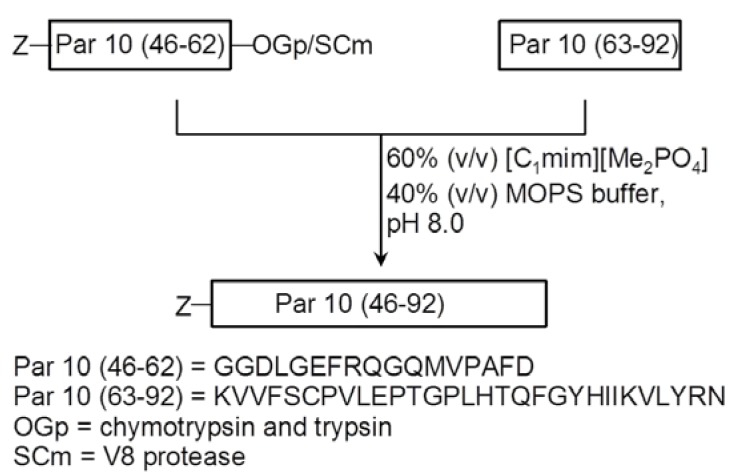
Protease-catalyzed ligation of parvulin (residues 46–92) employing peptidyl prolylcis/trans isomerase (modified from [48]).

In section 2.1.1, we described examples of thermolysin- and α-chymotrypsin-catalyzed couplings of amino acids to generate short peptides. Bordusa and co-workers, however, highlighted the protease-catalyzed ligation using ILs for the first time in 2008 [48]. The reaction was performed in a mixture of buffer and 1,3-dimethylimidazolium dimethylphosphate [C_1_C_1_IM][Me_2_PO_4_] (40:60) (Figure 8). Several positive effects were observed for this reaction in the IL mixture in comparison to conventional methods, e.g., (i) the complete suppression of proteolytic side reactions (proteolytic hydrolysis and decreases competing hydrolysis activity of proteases toward the acyl donor esters to some extent), (ii) high turnover rates, (iii) protease stability, and (iv) good solubility of the reactants and also of the reaction products.

**Figure 9:**
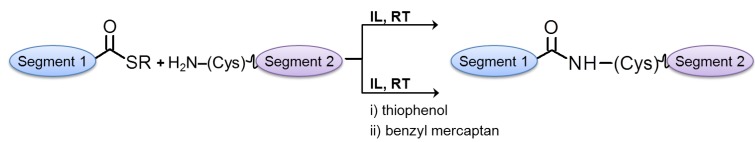
Native chemical ligation performed in ILs (modified from [78]).

Recently, we reported on the application of native chemical ligation to produce a 66-mer anticoagulant cysteine-rich peptide (Tridegin) that has been shown to act as an inhibitor of factor XIIIa (Figure 9) [78]. Generally, NCL may be carried out with and without additives, *i.e.*, thiols (e.g., thiophenol, benzyl mercaptan) as catalysts [79,80,81,82]. Therefore, the comparison of segment ligation in buffer and ILs in the presence or absence of additives was performed as well. The results showed that NCL indeed yields the ligated product. The linear Tridegin was identified in [C_2_mim][OAc] even in the absence of thiophenol and benzyl mercaptan. In addition, by using additives the ligated product was also formed in [C_2_mim][DEP] that correlates well with the results published by Bordusa and co-workers mentioned above [48].

In summary, the results of our and other groups demonstrate that medium-sized and large disulfide-bridged peptides are correctly formed in biocompatible ionic liquids. Moreover, the selection of peptide candidates examined thus far suggests that the correct intramolecular hydrogen bond interactions are not significantly perturbed by the presence of ILs, instead ILs seem to have a favorable effect on stabilizing pre-formed secondary structures of peptides.

## 3. Ionic Liquids for Peptide Purification and Characterization

Peptide purification and chemical characterization are the most important steps after a peptide has been synthesized. Usually peptides are purified using reversed-phase high performance liquid chromatography (RP HPLC) and characterized by different methods such as mass spectrometry (ESI or MALDI MS), thin-layer chromatography (TLC), and capillary electrophoresis. The structure of peptides can be determined by NMR spectroscopy. In this section, we give an overview of the applications of ILs in those methods, in which ILs have been applied so far.

### 3.1. Peptide Separation by Liquid Chromatography Methods

The use of ILs for chromatography reveals promising perspectives for the establishment of new separation methods and protocols. Examples for the application of ILs in separation techniques are well summarized in the review of Han *et al*. [83], but only few reports are found for the application in peptide chemistry. This field of investigation is rather new, however, the academic interest has increased enormously. Here, we explicitly highlight new applications of ILs for peptide separation and characterization, which were established in the last few years.

#### 3.1.1. Reversed-Phase HPLC

HPLC is a widely used method for separation and purification of biomolecules. Especially for peptide analysis RP HPLC is the most common method applied. Some reports have been published about the application of ILs in RP HPLC for separation of nucleotides, geometric isomers, ephedrines or carboxylic acids [84,85,86,87]. These reports are based on the use of ILs as additives for the mobile phase [84] or as a stationary phase [87]. The commonly used ILs for these processes contained 1,3-dialkylimidazolium or *N*-alkylpyridinium cations and chloride, tetrafluoroborate, hexafluorophosphate, or trifluoroacetate as the anion depending on the analyte [86]. Nevertheless, none of the reports represents an example of an IL-supported optimal separation of peptides. Marszall *et al*. suggested that it is not possible to find a suitable IL for separation and purification processes [88] according to the principle, which can be expressed by the basic chemical aphorism “similis similibus solvuntur” or “similar dissolves in similar”. Therefore, when the sample, *i.e.*, the peptide, is polar, the use of a polar IL with chaotropic anions, such as hexafluorophosphate or perchlorate was proposed, while a less polar IL with a cosmotropic anion (e.g., chloride) was recommended for a nonpolar analyte [86]. That is exactly why an individual protocol for different analytes is required.

##### 3.1.1.1. ILs as Additives for RP HPLC Mobile Phase

Ionic liquids have been used as additives to mobile phases in RP HPLC [86], because the relatively high viscosity of some ILs prevents their usage as pure eluent, in contrast to standard organic solvents (MeOH, ACN) commonly used for peptide purification. However, in the work of Polyakova *et al*., IL concentrations of 2–50 mM resulted in an improvement in the peak shape and decreased peak tailing or band broadening [84]. They use the separation of *o*-, *m*- and *p*-amino benzoic acid involving [C_2_mim][BF_4_], [C_4_mim][BF_4_], [C_2_mim][MeOSO_3_] and [C_8_mim][MeOSO_3_] as additives. The best separation was found with the methylsulfate systems and a concentration of 1.0~8.0 mM/L. The kinetics of the solute-stationary phase is responsible for this feature. Later, this group also reported on changes in the retention time of analyte elution, which decreased or increased depending on the type of IL used, *i.e.*, on the solute-stationary phase interactions [85], When the IL ions coat the silanol groups of the silica stationary phase the interactions of the analytes with the hydroxyl groups of the silica phase are minimized. Thus, the analyte eluted earlier [86]. Different models were proposed to explain this behavior: (1) ion-pairing, (2) adsorption of an IL layer on the C18 surface, or (3) linear solvation free energy relationship (LSER) [89,90]. The latter model was developed to describe the retention behavior in RP chromatography and included the calculation of intermolecular interactions [89,90].

##### 3.1.1.2. ILs as Material for RP HPLC Stationary Phase

**Figure 10:**
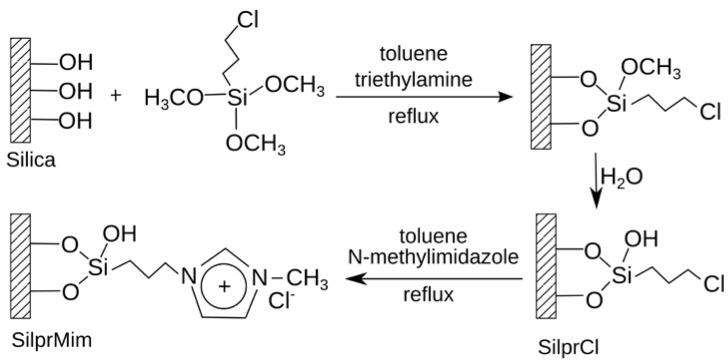
Procedure for the preparation of a *N*-methylimidazolium-functionalized silica gel stationary phase (modified from [92]).

Similar to the use of ILs as mobile phase for RP HPLC, researchers tried to use ILs as stationary phase. For example, the ionic liquid cation was covalently attached to the silica substrates by means of a linker, e.g., 3-mercaptopropylsilane or propyl [86,91]. Such stationary phases were named “surface-confined ionic liquids (SCIL)” (Figure 10) [91,92]. 

The commonly used ILs for this purpose are based on 1,3-dialkylimidazolium or *N*-alkylpyridinium cations with variably associated anions [91,93]. In 2010, the first example of SCIL applied to the separation of small peptides was reported by Chitta *et al*. [87]. They used a dipeptide (GY), a tripeptide (VYV), two pentapeptides (neuropeptides Leu-enkephalin, YGGPL, and Met-enkephalin, YGGPM), and an octapeptide (angiotensin II, DRVYIHPF) as subjects of investigation. The silica surface was modified with *n*-butylimidazolium bromide, and the effect of trifluoroacetic acid as ion-pairing agent was evaluated. In RP-HPLC of peptides, ion-pairing agents are commonly used as mobile phase additives. Such agents are primarily selected depending on the charge of the peptide(s) to be separated. Several volatile perfluorinated acids, such as TFA and pentafluoropropionic acid, are generally applied in this respect. They affect the retention behavior of a peptide through interactions of the ions with charged functional groups of the peptide’s side chains, e.g., of basic residues Lys, Arg and His, as well as of the N-terminal amino group. In turn, hydrophilicity of the peptides is reduced, since net charge is reduced and thus, ion-pairing ions influence the hydrophobic character of a peptide resulting in increased hydrophobic interactions with the reversed-phase sorbent [87]. However, TFA in different concentrations (0.001–0.1% v/v) did not act as ion-pairing agent for the elution systems used by Chitta *et al*. and thus, can be substituted by formic acid in these systems. Although the SCIL phase possesses reversed-phase character, it was found that electrostatic interactions dominated at high organic and/or low pH modifier concentrations [87]. 

#### 3.1.2. Thin Layer Chromatography

**Figure 11:**
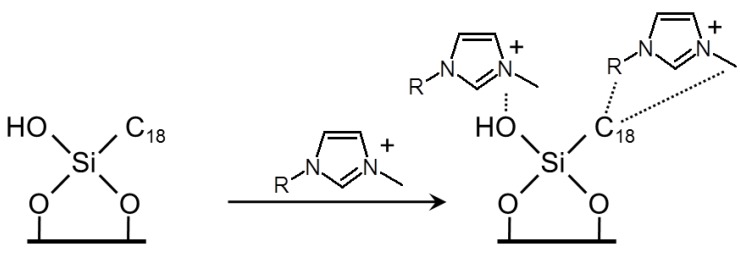
Schematic representation of a silica-based stationary phase interacting with an imidazolium-based IL, where -R represents alkyl substitutions (proposed by He* et al*.) (modified from [96]).

Ionic liquids were not only applied to RP HPLC, but also used for TLC and normal phase HPLC [94,95]. However, characterization of basic or amphoteric samples is challenging if using these methods. The problem is that acidic silanol groups of the stationary phase interact strongly with organic bases (Figure 11) resulting in (i) an increase in retention, and (ii) a broadening and/or tailing of sample peaks [96].

It has already been reported that the use of IL additives may help to overcome these problems. Ionic liquids block silanol groups and in this way provide the desired separation of basic drugs as test analyte [97]. Similarly to RP HPLC, alkyl substituted imidazolium ILs were used for TLC and HPLC as well. Baczek *et al*. investigated the effect of the percentage amount (0–10%) of the ILs as additives to the eluent on the separation of peptides by TLC. For approximately 50 peptides the R_f_ values increased with an increased concentration of 1,3-dimethylimidazolium methyl sulfate in a water/0%–5% acetonitrile/0.1% TFA mixture [98]. The influence on the retention of the peptides with an increasing IL concentration was weaker with increasing length of the amino acid sequences. In the study of Baczek *et al*., 1-ethyl-3-methylimidazolium tetrafluoroborate was applied. Upon addition of acetonitrile (1.5%) a retention coefficient with third-degree polynomial function was derived, however, a quadratic function was needed in case of the experiment without acetonitrile. This was explained by the interaction of the IL with the stationary phase [99].

Another method involved the addition of IL and matrix (α-cyano-4-hydroxycinnamic acid) to the separation mixture. This optimized the chromatographic method allowing for a direct coupling of the thin-layer chromatograms with imaging by MALDI mass spectrometry (see also section 3.2) [100].

### 3.2. Mass Spectrometry

MALDI mass spectrometry is a widely used method for the characterization of biomolecules [101,102] as individual components from mixtures or even whole tissues [16]. The main characteristic of this method is the “soft” ionization in comparison to other methods, e.g., electrospray ionization [103].

For successful analytical results a suitable matrix is indispensable. Matrices absorb the energy of the laser and support ionization of the analyte. They are usually small acidic compounds that co-crystallize with analyte molecules and provide the access of protons [15,104]. Main problems of standard solid matrices are low homogeneity, formation of so called “hot spots” and/or adducts of the analyte with cations and anions. This results in low measurement reproducibility, decreased sensitivity, broadening or suppression of peaks [105]. Commonly used matrices for MALDI MS of peptides and proteins are α-cyano-4-hydroxycinnamic acid (CCA), sinapinic acid (SA), 2,5-dihydroxybenzoic acid (DHB), and indole acrylic acid.

ILs as matrices for peptide analysis with MALDI mass spectrometry were first reported in 2004 by Mank *et al*. [106]. Different combinations of matrices were probed, but the CCA-butylamine (1:1) was described to be the best choice for MALDI MS of the peptides investigated. One of the reasons was that alkali adducts with the peptides were observed if the DHB-butylamine (1:1) matrix was applied, which was prepared in an ethanolic ionic liquid 1:1 (v/v) mixture. Herein, it was shown for the first time that this DHB-IL matrix can be used for a direct and easy screening of enzymatic reactions [106]. In the same year, another report of Zabet-Moghaddam *et al*. was published, where equimolar mixtures of SA, CCA, and DHB with organic bases, such as tributylamine, 1-methylimidazole or pyridine were used [105]. It was shown that ILs were not suitable as MALDI matrices without addition of classical matrices. The best results were achieved for the use of water immiscible ILs, e.g., [C_4_mim][NTf_2_] or [C_4_mim][PF_6_], with the addition of CCA. At the same time, peptides behaved differently in the IL-matrix compared to the IL-free medium [105]. This fact was also mentioned in later research works [103], stating that the homogeneity of the samples decreased in comparison to conventional matrices [105]. However, these first examples on the use of IL-mixtures with classical matrices for peptide analysis demonstrated an improved spot-to-spot reproducibility and better ionization efficiency. Since then, numerous reports appeared in the literature aiming to find out the best matrix system for peptide analysis. Many different combinations of the basic part of an IL and the acidic part of the standard matrices were used [107]. From that time on, these mixtures are called “ionic liquid matrices (ILM)” [103]. Jones *et al*. also showed an enhanced sensitivity of the MALDI TOF method for peptide characterization if such ILM were used [103]. However, the first reports of the analysis of synthetic peptides with ILMs were described in 2006. A reduction of peptide adduct and matrix cluster formation, an improvement of the signal-to-noise ratio and an increase of the peak intensities was observed for the use of a CCA-pyridine ILM (2:1, v/v). Also, the detection limits were reduced using the described ratio [108]. In addition, an improvement in sample homogeneity was reported by Tholey *et al*. [109] who showed that the quantitative analysis of peptides was possible without the use of an internal standard. Additionally, a linear correlation between the peptide amount and the signal intensities was observed. Here, the samples were prepared with the ILM in a matrix(acid)-IL(base) ratio of 1:1. As bases, *N*,*N*-dimethylethylenediamine (DMED) and 3-(dimethylamino)-1-propylamine were used, and as matrix CCA and indole acrylic acid were applied. Both matrices were found to be suitable for peptide analysis. The addition of a matrix additive (1% H_3_PO_4_) to increase the detection limit and signal intensities was reported by the same research group [110]. Phosphopeptides were used as targets for the MALDI MS analysis described.

A new way of sample preparation for high sensitivity quantitative analysis of peptides was proposed by Palmblad *et al*. [111]. A conductive hydrophobic Teflon^TM^ tape was used as sample surface in the analysis in order to concentrate the analyte to microliter range and to achieve better ionization. Also, the successful attachment of cations (Cu^2+^ and K^+^) to target peptide angiotensin I in the ILM CCA/3-aminoquinoline/glycerol (1:4:6) in comparison to the standard dried droplet method and powder mixture sample preparation was reported by Hortal *et al*. shortly after [112]. In 2008, a further ILM was proposed as suitable matrix for peptide analysis with MALDI mass spectrometry, which was a mixture of CCA and aniline. This matrix was also structurally characterized. A similar or even improved signal-to-noise ratio was observed in comparison to the pure CCA matrix [113]. Other ILMs such as *N*,*N*-diisopropylethylammonium-CCA and *N*-isopropyl-*N*-methyl-l-butylammonium-CCA matrices were reported to be very suitable for the analysis of peptides and proteins in the molecular weight range from 1 kDa to 270 kDa. For example, these matrices showed much higher intensities of the analyte [114].

The efforts to use ILMs for the analysis of glycopeptides and glycans succeeded when using the matrix [1,1,3,3,-tetramethylguanidinium-2,4,6,-trihydroxyacetophenone] (GTHAP). The detection of glycopeptides containing large carbohydrate groups and small peptide chains was higher than in the solid matrix (2,4,6,-trihydroxyacetophenone). In addition, the degree of glycopeptide decomposition was suppressed if using GTHAP [115]. The avoidance of sugar fragmentation as well as the improvement of homogeneity and reproducibility of the spots were also important issues in the report of Gimenez *et al*., who investigated the effectiveness of different ILMs with SA and DHB on the ionization of intact glycoproteins with several degrees of glycosylation [116]. All ILMs preparation procedures used butylamine as organic base and were prepared either with or without an acid, e.g., 0.1% of TFA or 1% of H_3_PO_4_. The authors demonstrated that the chemical composition of an ILM strongly influenced the analysis of their test substances. In addition, ionization efficiencies and spot homogeneity were best if using ILMs with higher amounts of the organic salt, *i.e.*, the ILM containing 3 M of butylamine and an equimolar amount of either DHB or SA was the optimal preparation procedure. The results for the average molecular mass values also indicated that sugar fragmentation was prevented for either mixture, while the SA-ILM was best with respect to reliable analysis, sensitivity, sharpness of ion peaks and application of a wider range of laser intensities [116]. Finally, new reports by Fitzgerald and co-workers showed that ILMs are suitable for MS imaging, e.g., of onion skin, as well [117]. Here, 1-methylimidazolium-CCA and tripropylammonium-CCA were applied as matrices. Both were suggested to improve the detection limits to about 30- to 40-times in comparison to the standard matrix. Again, homogeneity and stability of the sample surface during the crystallization process was improved [117].

One further application of ILs in mass spectrometry other than the MALDI technique should be mentioned here as well, where ionic liquids were used for the identification of integral membrane proteins (IMPs) by microcolumn reversed phase liquid chromatography (μRPLC)-electrospray ionization tandem mass spectrometry (ESI-MS/MS) [118]. The authors here used 1% (v/v) [C_4_mim][BF_4_] in NH_4_(HCO_3_) buffer (pH 8.3) as the sample preparation buffer for IMPs analysis. Furthermore, compared to the commonly used methods (e.g., sodium dodecyl sulfate and methanol) a three times higher number of identified IMPs from rat brain was obtained, which was explained by the fact that [C_4_mim][BF_4_] may provide higher solubilizing ability for IMPs and good compatibility for tryptic digestion. 

## 3. Conclusions

The application of ionic liquids in peptide chemistry is currently in the exciting phase of its development. The assembly of peptides by SPPS or in solution using ILs has been described, but primarily for shorter representatives. Therefore, these investigations are, in our opinion, in an early stage of development. Nevertheless researchers continue to find optimal more simple and straightforward conditions to carry out reactions involving amino acid derivatives and peptides. In general, synthesis and characterization of peptides using ionic liquids seems to have several positive effects, including the better solubility of solutes, the use of higher temperature, the possibility to reduce the concentrations of excessive agents, higher reaction yields, and higher resolution (analytics). 

It has been shown for synthetic processes that ILs are not just simple solvents, but may act as a functional part in a reaction process, e.g., by directly interacting with analytes. In addition, many reports concerning the characterization of peptides by mass spectrometry, in particular MALDI-TOF MS, and different types of chromatography were published in the last decade. On the other hand, methods such as peptide/protein structure determination by NMR spectroscopy or separation by capillary electrophoresis are not as popular in the literature, yet are starting to gradually emerge. The reason for this may be that a deeper understanding of the interactions of IL cations and anions with specific side chains and/or the peptidic backbone in dependence on the IL environment and the absence and/or presence of water is still missing. Thus, a closer examination of these interactions would support both, the design of suitable ILs for special applications concerning the generation of a peptide (primary amino acid sequence) and/or its conformation (three-dimensional structure) and the optimization of purification and characterization protocols. Future studies will therefore focus on intensive and systematic investigations in order to address the questions raised by the early examples summarized in the present report.
